# Acute diverticulitis requiring hospitalization according to regional discrepancies in France between 2013 and 2022: a nationwide study

**DOI:** 10.1007/s00423-024-03536-0

**Published:** 2024-11-08

**Authors:** C. Saint-Dizier, J. F. Hamel, A. Lamer, A. Venara, M. Levaillant, Aurélien Venara

**Affiliations:** 1 Fédération Régionale de Recherche en Psychiatrie et Santé Mentale (F2RSM Psy), Hauts-de-France, Saint-André-Lez-Lille, France; 2 Univ. Lille, CHU Lille, ULR 2694-METRICS, Évaluation des Technologies de Santé et des Pratiques Médicales, Lille, France; 3 Faculté de Santé, Département de Médecine, Université d’Angers, Angers, France; 4 Département de Biostatistique, CHU Angers, 4 rue Larrey, Angers, 49933 France; 5 Service de Chirurgie Viscérale et Endocrinienne, CHU Angers, 4 rue Larrey, Angers, 49933 France; 6 The Enteric Nervous System in Gut and Brain Disorders, Nantes Université, CHU Nantes, INSERM, IMAD, Nantes, France; 7 Department of Visceral Surgery, CHU Angers (Angers University Hospital), 4 rue Larrey, Angers Cedex 09, 49933 France

**Keywords:** Diverticulitis, Ecological parameters, Nationwide, Surgery

## Abstract

**Purpose:**

The prevention of colon diverticulitis tends to be tailored according to the patients. In order to improve the public health policy to prevent diverticulitis, the influence of regional parameters at a department scale has to be assessed.

**Objective:**

This analysis aimed to assess the occurrence of acute diverticulitis in France in general and according to environmental factors suspected to affect such diseases.

**Methods:**

All patients above 18 years old admitted to a general hospital with a diverticulitis diagnosis between 2013 and 2022 in France were included. Data were extracted from the French national hospital discharge database. The primary outcome was the occurrence of diverticulitis according to French territories and known risk factors.

**Results:**

In this nationwide cohort study, the 10-years cumulative occurrence of diverticulitis in France was 3.45% (*n* = 2 0.248.099 patients). Diverticulitis was influenced by older age and male gender but was not significantly associated with ecological parameters (obesity, alcohol consumption, smoking or economic discrepancies) at a departmental scale. Of all patients diagnosed with diverticulitis, 5% had at least one surgical intervention. The surgical management of diverticulitis was associated with an increased number of surgeons in the department, even after adjustment for age and sex.

**Conclusions:**

Except for smoking, the frequency of diverticulitis requiring an hospitalization was independent of regional parameters (nor alcohol intake, nor obesity nor the economic discrepancies).

**Supplementary Information:**

The online version contains supplementary material available at 10.1007/s00423-024-03536-0.

## What does the study add to the literature?

The risk of diverticulitis depends on the patients risk factors but the influence of ecological parameters at a departmental scale is unknown. This study supports that, to the exception of smocking, such parameters (obesity, alcohol consumption or economic discrepancies) are not associated with at a departmental scale.

**Supplementary Information:**

The online version contains supplementary material available at 10.1007/s00423-024-03536-0.

## Introduction

The lifetime risk of diverticulitis was reported to range from 10 to 25% for persons with diverticulosis [1] but this rate may be overestimated as a more recent study highlighted a risk of 1.7% of diverticulitis in patients with diverticulosis followed over a period of 5 years [2]. Furthermore, the lifetime risk of hospitalization is estimated to be between 3 and 5% [3].

Diverticulosis affects up to 65% of people older than 80 [4] and its prevalence is rising in western countries and Japan [5]. Public health policies to prevent diverticulitis are not easy to apply as the first risk factor for diverticulitis is the presence of diverticulosis, which is mainly symptomless. Such features cannot be known apart from invasive or expensive examinations such as colonoscopy or CT-scan.

It would be advisable that public health policy focus on preventing the recurrence of diverticulitis because the risk of such a relapse episode is estimated at around 20% [1, 6].

To reduce the risk of recurrence and the risk of complicated diverticulitis, colon resection was historically recommended after 2 episodes of diverticulitis without considering the patient’s quality of life [7]. However, such colon resections were associated with long-term bowel dysfunction [8], with a risk of recurrence from 6 to 15% within 5 years [9] and with a risk of surgical morbidity and especially of stoma in 10% [9, 10]. More recent recommendations are focused on the patients’ decision, targeting an improvement of their quality of life [9] as elective sigmoid resection with a good indication improves the quality of life over conservative treatment with 2 years [11]. A recent study on the practices of American surgeons reveals that most have discarded older dogma [12]. Objectives are to focus on shared decisions between surgeons and patients on education or communication-based interventions [6, 12].

These changes in patient management require better knowledge about factors associated with recurrence to improve the discussion and decision-making with patients regarding the need and timing of elective surgery [13]. Diverticulosis is considered to be multifactorial, including genetics and environmental factors [14] but this has not been assessed on a country-wide scale.

This analysis aimed to assess the occurrence of in-hospital acute diverticulitis in France as well as the distribution across French departments. A second objective of this study was to assess the occurrence of diverticulitis according to environmental factors suspected to affect such diseases as the number of visceral surgeons, the ratio of smokers, the scale of alcohol consumption and socioeconomic discrepancies. Finally, our study explored the surgical management of such diverticulitis, according to the same features as described previously.

## Materials and methods

We conducted a historical cohort study on the French national hospital discharge database (PMSI; Programme de Médicalisation des Systèmes d’Information). We followed the French reference methodology MR-005 of the Commission Nationale de l’Informatique et des Libertés (the French Data Protection Authority), which provides a framework for access to data from the PMSI, by healthcare institutions and hospital federations. This database is made available on the secure platform of the French technical agency for information on hospitalization (Agence technique de l’information sur l’hospitalisation). As the data was not available at an individual scale but at a departmental scale, there was no individual properly included in the study. For this reason, no consent was required.

### Data source

The PMSI database collects individual-level data about the patients and their hospital stays, age, sex and place of residence. All inpatient stays in public and private hospitals are described with diagnoses, medical acts, date of admission, length of stay, hospital code number, hospitalization category, and outcome (i.e., discharge, hospital transfer, death). Diagnoses were documented according to the French version of the International Statistical Classification of Diseases and Related Health Problems, 10th Revision (ICD-10). Medical acts are documented with the French terminology of the *Classification commune des actes médicaux* (CCAM).

This medico-administrative database does not contain any valuable information about clinical results. Data are fully anonymized.

The frequency of tobacco consumption and obesity in French departments (e.g. French administrative territorial unit) could be extracted from the “State of health of the French population” study conducted by the DREES (Direction de la recherche, des études, de l’évaluation et des statistiques) [15].

No data describing the frequency of alcohol consumption in the French departments could be found, but the incidence ratios (standardized on French mortality during the 2007–2014 period) of cancers specifically linked to alcohol could be extracted from the National cancer institute website [16]. Considered cancers were lips, mouth, pharynx, esophagus and larynx cancers.

The number of surgeons could be extracted from the Annual statistics of health establishments database [17].

### Study population and data collected

All patients above 18 years old admitted to a general hospital with a diverticulitis diagnosis [ICD-10 code K57.3] between 2013 and 2022 were included. We extracted data from all the corresponding hospital stays.

Variables collected in the PMSI were age (above or below 60 years old within the first diagnosis), sex, department of residence, hospital in which the stay took place, surgical procedure for the treatment of diverticulitis (identified with the CCAM codes HHFA002, HHFA006, HHFA010, HHFA014, HHFA017, HHFA024, HHFC040, HHFA008, HHFA009, HHFA026, HHFC296, supplementary Table 1). Diagnosis and procedure codes were selected based on the physician’s practice and based on the French Technical Agency for Information on Hospitalization. Data were completed by an ecologic analysis using a departmental scale [18]. The unit of the observations in the model is the French department. The French department denotes an administrative French territorial unit.

The primary outcome was the occurrence of diverticulitis according to French territories and known risk factors. The secondary outcome was the rate of surgically managed diverticulitis according to the ratio of surgeons per 100,000 inhabitants.

### Statistical analysis

Frequency of diverticulitis occurrence was described using cumulative occurrence over the 10 years study period. The relationship between diverticulitis and its potential risk factors was studied using mixed linear regressions, considering French departments as a random effect covariate. As data were collected at the department level, a sampling weight corresponding to the department population was applied using the Graubard and Korn method [19]. The potential diverticulitis risk factors considered in this study were gender ratio, age ratio (59 and younger vs. 60 and older), rate of smokers, rate of obesity, occurrence ratios of alcohol related cancer in the department, socio-economic factors (Gini index, unemployment rate and income support beneficiary rate) and departmental rate of surgeons. Validity of the considered models were verified through residuals analysis. All the tests were performed using a type-I error threshold set at 0.05.

## Results

### Occurrence of in-hospital diverticulitis in France

Between 2013 and 2022, 2,248,099 patients were identified as having diverticulitis, leading to a 10-year cumulative occurrence of 3.45% in France. The cumulative occurrence of diverticulitis was higher for patients older than 60 compared to patients aged 59 and younger (9.3% vs. 1.3%, *p* < 10^− 4^). The 10-year cumulative occurrence of diverticulitis was 3.4% for females and 3.5% for males. A significant interaction between gender and age was found (*p* < 10^− 4^). The occurrence of diverticulitis was lower in the group of older women than in the group of older men (8.7% versus 10.1%, *p* < 10^− 4^).

### Occurrence of in-hospital diverticulitis among French territories

**Fig. 1:**
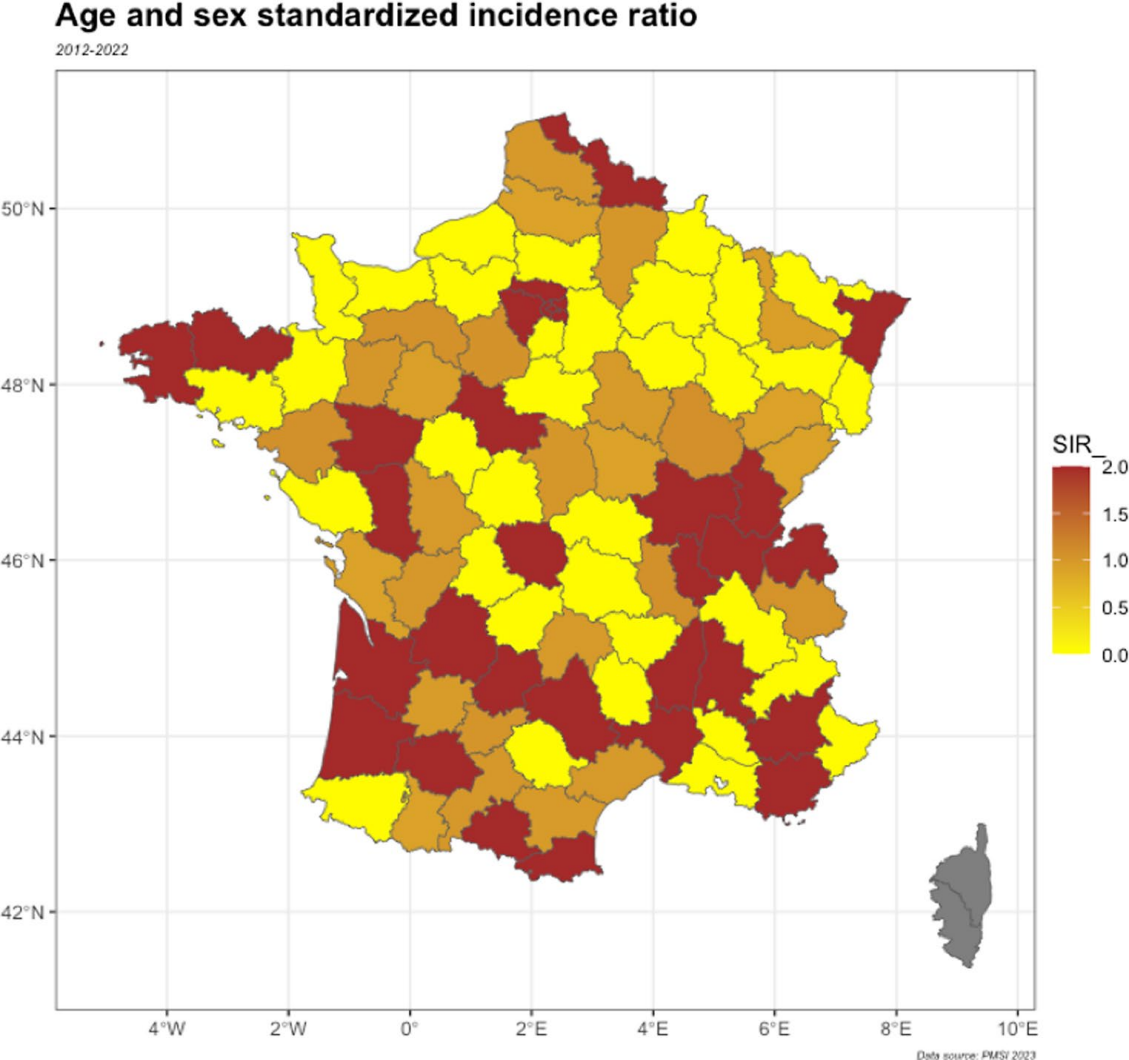
Age and sex standardized incidence ratio of diverticulitis among French departments

Cumulative occurrence varied among French departments, from 1.27% in the lowest one to 6.00% in the highest (SD = 0.01) (Fig. 1).

### Impact of known risk factors on the cumulative occurrence of diverticulitis among French departments

Ten-year cumulative occurrence was significantly associated with the rate of smokers in the department (*p* < 0.001) but not with the obesity rate (*p* = 0.32) nor with the rate of alcohol use-induced cancer (*p* = 0.11) (Table 1). For every one-percent increase in the smoking rate in the department, the cumulative occurrence of diverticulitis rises by 0.1% (*p* < 0.001) (Table 1). Occurrence was not associated with the socio-economic factors studied, namely the Gini index (*p* = 0.969), unemployment rate (*p* = 0.859) and income support beneficiary rate (*p* = 0.655).

**Table 1:** Linear regression of factors associated with at least one episode of diverticulitis among French territories

	Coef.	[95% Conf. Interval]		*P* > z
Age above 60 years old	0.080	0.076	0.085	0.000
Female gender	-0.008	-0.009	-0.007	0.000
Smokers	0.001	0.000	0.002	0.044
BMI > 25	-0.004	-0.012	0.004	0.316
Alcohol induced cancers	0.004	-0.001	0.008	0.115

### Relationship between surgical management of diverticulitis and the number of surgeons in each department

Of all patients diagnosed with diverticulitis, 5% had at least one surgical intervention.

**Fig. 2:**
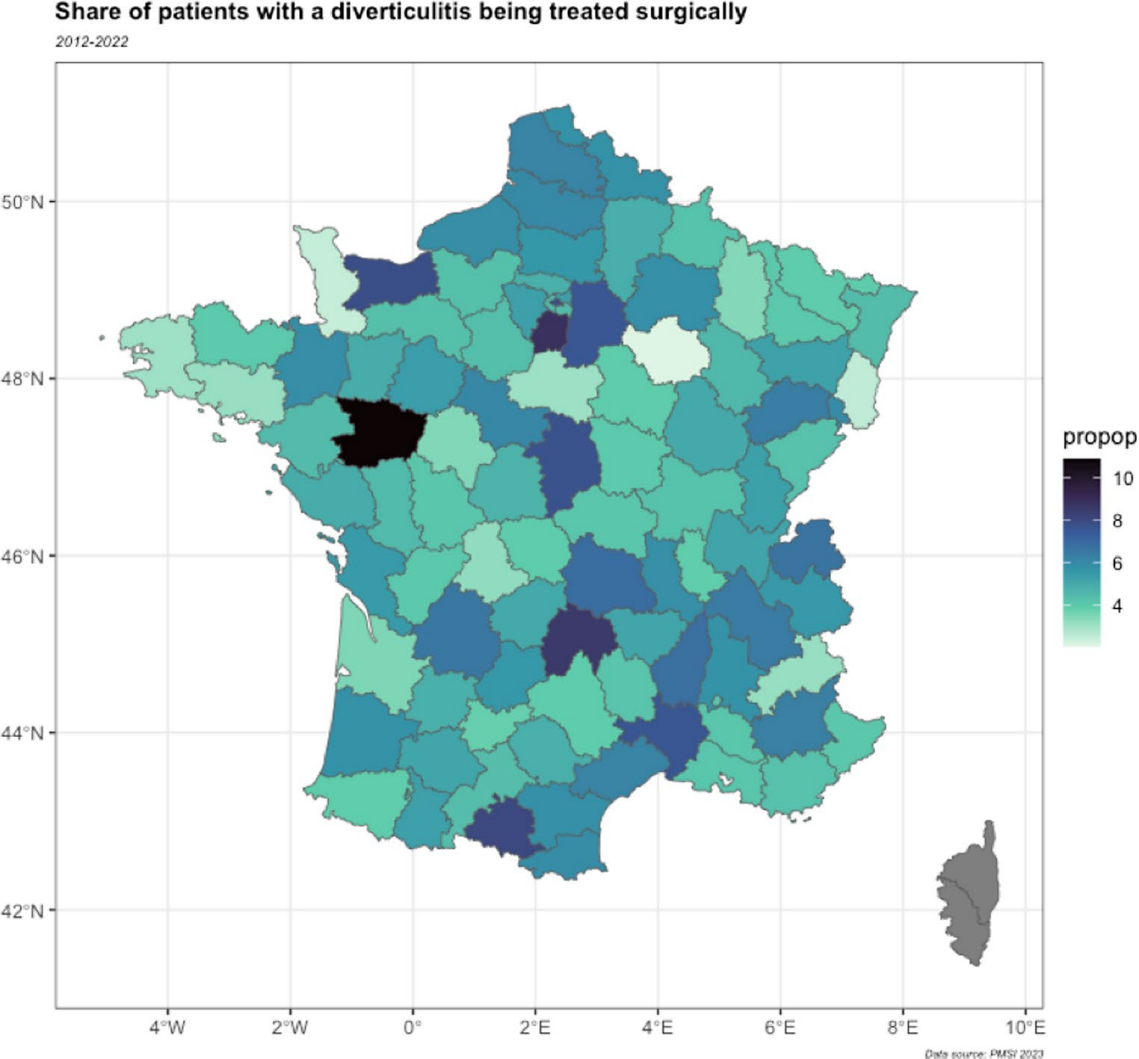
Rate of surgically treated patients with diverticulitis among French departments

The rate of surgical treatment was lower for patients aged 60 and above than in younger ones (4.2% vs. 5.3%, *p* < 10^− 4^). The repartition of diverticulitis and surgical management according to the gender is reported in Table 2. The surgical rate was higher for men than for women (5.5% vs. 4.6%, *p* < 10^− 4^). A significant interaction was found between gender and age (*p* < 10^− 4^). Surgical rates between age and sex are presented in Table 3. The surgical rate varied among French departments, from 2.1% in the lowest one to 10.9% in the highest (SD = 0.02) (Fig. 2).

**Table 2:** Number of diverticulitis and surgical management according to the gender of patients

	Male	Female
Number of inhabitants	31 605 641	33 663 513
Number of patients non operated	1 065 228	1 082 422
Number of patients operated	51 556	49 792

**Table 3:** Number of diverticulitis and surgical management according to the age of patients and rate of treated at least once surgically among patients with diverticulitis between 2013 and 2022 in France according to age and sex

	Below 60 years old	60 years old and older
Number of inhabitants	47 816 124	17 453 030
Number of patients non operated	588 893	1 558 757
Number of patients operated	32 809	68 539
Rate of patients treated at least once surgically		
*Men*	5.93%	4.11%
*Women*	4.70%	4.35%

Interaction between age and sex was found significant (*p* < 10^− 4^)

**Fig. 3:**
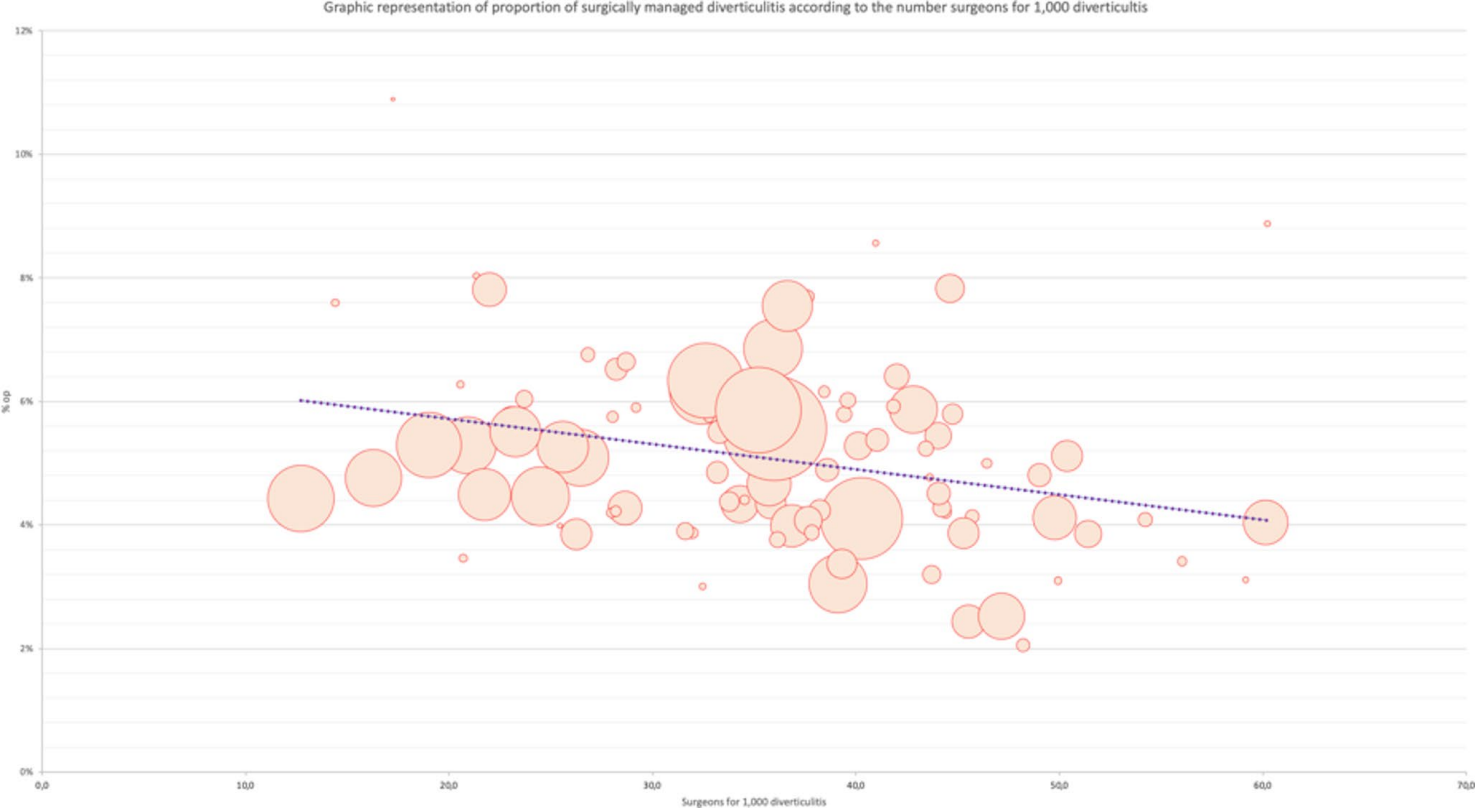
Relationship between surgeon number per 100,000 inhabitants and surgically managed diverticulitis

A high number of surgeons in the department increased the risk of surgical management; for each increase of 1 surgeon per 100,000 inhabitants, the rate of diverticulitis treated surgically increases by 0.16% (*p* = 0.01). Such relation between surgeon number and surgical rate was significant after adjustment on the gender ratio and age of patients with diverticulitis in each department, with a coefficient of 0.2% (*p* < 10^− 4^) (Table 3; Fig. 3) (See Table 4 and 5).

**Table 4:** Linear regression of factors associated with a surgically managed diverticulitis among French territories

	Coef.	[95% Conf.	Interval]	*P* > z
Number of surgeons per 100,000 inhabitants	0,002	0,000	0,003	0,013
Age above 60 years old	-0,010	-0,013	-0,007	0,000
Female gender	-0,005	-0,006	-0,004	0,000

**Table 5:** Linear regression of factors associated with surgical management of diverticulitis according to known risk factors among French territories

	Coef.	[95% Conf.	Interval]	*P* > z
Number of surgeons per 100.000 inhabitants	0.002	0.001	0.004	0.001
Age above 60 years old	-0.010	-0.012	-0.007	< 0.001
Female gender	-0.005	-0.006	-0.004	< 0.001
Smokers	0.001	0.000	0.002	0.18
IMC > 25	0.007	-0.001	0.014	0.08
Alcohol induced cancers	0.003	0.000	0.005	0.07

As for the relationship between surgical rate and the known risk factors, in a multivariate analysis, such relation was significant after adjustment on the gender ratio and age of patients with diverticulitis in each department, with a coefficient of 0.2% (*p* < 10^− 4^).

## Discussion

In this nationwide cohort study, the 10-years cumulative occurrence of diverticulitis in France was 3.45%. Diverticulitis was influenced by older age and the male gender but was not significantly associated with ecological parameters at a departmental scale. The surgical management of diverticulitis was associated with an increased number of surgeons in the department, even after adjustment for age and sex.

First, the occurrence of diverticulitis in France was similar to data reported in the literature. Indeed, literature reports that the prevalence of diverticulosis in Western countries is 5–45% and that the risk of complications of a diverticulosis during a lifetime is 4–15% [20]. Moreover, a recent review article reports that the risk of hospitalization for diverticulitis episodes increases from 74.1 per 100 000 habitants in 2000 to 91.9 per 100 000 habitants in 2010 [20]. The documentation of the episodes of diverticulitis in France can therefore be considered reliable and can allow a further analysis of factors associated with such episodes.

Interestingly, the factors associated with diverticulitis were not similar with the literature as literature reports that the risk of diverticulitis is higher in women than in men [20, 21]. Moreover, the prevalence of diverticulosis increased with age [15, 22] which leads to an increase in the prevalence of diverticulitis with age [15, 17]. Except for smoking, our study did not find ecologic parameters associated with a cumulative occurrence of diverticulitis while literature suggests an association with alcohol intake, smoking and obesity [20, 22]. This can be explained by the ecological design of our study, for which individual characteristics should not be deduced from inferences about the group to which those individuals belong [23, 24].

However, our study aimed to assess the role of ecological characteristics at a departmental level toward in diverticulitis occurrence and surgical management. To date, the best treatment strategy for recurrent diverticulitis has not been identified [25]. More detailed knowledge of the risk factors at a departmental scale should enable to adapt public health policies to reduce the risk of occurrence and improve the day-to-day lives of the patients concerned. Indeed, although our study provides data on the potential impact of gender, age and tobacco consumption on the occurrence of diverticulitis, a difference in occurrence remains between French departments. Such geographical variation may reflect an unidentified environmental factor on the occurrence of diverticulitis, such as dietary habits [1, 26, 27], climate [28] or reflect an infectious etiological aspect.

The surgical management was associated with male gender and with age < 60 years. This is in accordance with the French recommendations for diverticulitis surgery [29]. Finally, the number of surgeons in the department appeared significantly associated with the surgical management of diverticulitis, even in the multivariate analysis. This last result can be interpreted following 2 possibilities: the surgeons are well distributed in France (e.g. the access to surgical management is good where the occurrence of diverticulitis is high) or the patients undergo surgery where the surgeons are (e.g. the patients move to the neighbor department to undergo surgery). The design of this study does not allow the correct supposition to be deduced. One other possibility is that indications are not equal over the French territory but the design of the study does not allow to study the indications for surgery.

This study presents however some biases that have to be considered. Indeed, only patients that were admitted to a general or a private hospital with diverticulitis were included; patients for whom diverticulitis was managed outside hospital were not taken into account and may include an underestimation of the occurrence of the episodes of diverticulitis managed conservatively. Also, as it is a medico-administrative database, no medical information (classification, clinical manifestation of the disease…) is available on the database and the severity the diverticulitis is not documented in the PMSI database. Also, being an analysis of a medico-administrative databases, it is not possible to make the quality assessment of the data over the databases used. However, these databases are used to pay the hospitals for their work according to the diagnoses of the patients. The diagnoses should therefore be well documented for the hospital to be paid as they should be and they should be well documented for the national health institute to pay correctly the hospitals. Random controls are provided by the National Health Institute to ensure the hospitals make the good coding. Also, it was not possible to be sure that the patients treated in a department were resident in that French department, representing a potential bias. Those, the study depicts the link between ecological parameters and in-hospital diverticulitis at a French department scale, independently where the patients live. Lastly, this database did not allow for analysis of the potential delays in management according to the accessibility of surgery in different departments. However, despite these biases, this study highlights the potential risk factors and ecological factors associated with diverticulitis and brings some evidence that could help to build tailored public health policy to reduce the cost of the burden of diverticulitis.

## Conclusion

The occurrence of diverticulitis increases in elderly and in male gender at a nationwide level. However, except for smoking, the frequency of diverticulitis requiring an hospitalization was independent of regional parameters (nor alcohol intake, nor obesity nor the economic discrepancies).

## Electronic supplementary material

Below is the link to the electronic supplementary material.

Supplementary Material 1

Supplementary Material 2

Supplementary Material 3

## Data Availability

No datasets were generated or analysed during the current study.

## Declarations

### Data sharing statement

Data are available for reasonable request by addressing an e-mail to the corresponding author.
