# Supplementary material for: Acute diverticulitis requiring hospitalization according to regional discrepancies in France between 2013 and 2022: a nationwide study
Source: Langenbecks Arch Surg. 2024 Nov 8;409(1):337. doi: 10.1007/s00423-024-03536-0 (PMC11549154; doi:10.1007/s00423-024-03536-0)
Supplement: Supplementary file 2 — Supplementary Material 2 [file 423_2024_3536_MOESM2_ESM.docx]

**HHFA002 : Left colectomy with release of the left colic flexure, with anastomosis, by laparoscopy or open surgery with preparation by laparoscopy**

**HHFA006 : Left colectomy with release of the left colic flexure, with anastomosis, in open surgery**

**HHFA010: Left colectomy without release of the left colic flexure, with anastomosis, by laparoscopy or open with preparation by laparoscopy**

**HHFA014: Left colectomy without release of the left colic flexure, without anastomosis, by open surgery**

**HHFA017: Left colectomy without release of the left colic flexure, with anastomosis, by open surgery**

**HHFA024: Left colectomy with release of the left colic flexure, without anastomosis, by open surgery**

**HHFC040: Left colectomy without release of the left colic flexure, without anastomosis, by laparoscopy**

**HHFA008: Right colectomy with anastomosis, by laparoscopy or open with preparation by laparoscopy**

**HHFA009: Right colectomy with anastomosis, by open surgery**

**HHFA026: Right colectomy without anastomosis, by open surgery**

**HHFC296:** **Right colectomy without anastomosis, by laparoscopy**
